# Immunomodulatory Potential of Human Mesenchymal Stem Cells and their Exosomes on Multiple Sclerosis

**DOI:** 10.34172/apb.2022.038

**Published:** 2021-02-06

**Authors:** Hussein Baharlooi, Zahra Salehi, Moein Minbashi Moeini, Nima Rezaei, Maryam Azimi

**Affiliations:** ^1^Department of Immunology, School of Medicine, Tehran University of Medical Sciences (TUMS), Tehran, Iran.; ^2^Students’ Scientific Center, Tehran University of Medical Sciences (TUMS), PO Box 1417755331, Tehran, Iran.; ^3^Department of Physiology, School of Medicine, Tehran University of Medical Sciences, Tehran, Iran.; ^4^Faculty of Pharmacy, Université Laval, Centre de recherche de l’Institut universitaire de cardiologie et de pneumologie de Québec (CRIUCPQ), Québec, Canada.; ^5^Research Center for Immunodeficiencies, Children’s Medical Center, Tehran University of Medical Sciences (TUMS), Tehran, Iran.; ^6^Network of Immunity in Infection, Malignancy and Autoimmunity (NIIMA), Universal Scientific Education and Research Network (USERN), Tehran, Iran.; ^7^Immunology Research Center, Institute of Immunology and Infectious Diseases, Iran University of Medical Sciences (IUMS), Tehran, Iran.

**Keywords:** Relapsing-remitting MS, Mesenchymal stem cell, Exosome, Immunomodulation

## Abstract

***Purpose:*** Promising advances have been made in mesenchymal stem cell transplantation to reinducethe immune tolerance in neuroinflammatory animal models and multiple sclerosis (MS)patients. The available evidence demonstrated that immunomodulatory effects of mesenchymalstem cell are particularly exerted through releasing exosomes to their environment. Wetherefore, aimed to comparatively assess the potential effect of mesenchymal stem cells andmesenchymal stem cells-derived exosomes on proliferation and function of the CD4^+^CD25^−^ conventional T cells, isolated from relapsing-remitting MS patients.

***
Methods:*** Mesenchymal stem cells were isolated from human umbilical cord tissues and usedfor exosome isolation via ultracentrifugation. Both mesenchymal stem cells and mesenchymalstem cells-derived exosomes were evaluated for their anti-inflammatory effects againstthe proliferation of T cells isolated from two groups of individuals *in vitro*, MS patients andhealthy subjects. Cytokine production of conventional T cells (interferon-γ, interleukin-10, andinterleukin-17) was also assessed, using flow cytometry for the patients and healthy individuals.

***
Results:*** Here, evidence shows that MSCs and MSC-derived exosomes dampen proliferationand percentage of conventional T cells that produce IFN-γ (healthy control: *P* < 0.001) andinterleukin-17 (healthy control: *P* <0.001, MS patients: *P* < 0.001), with a significant increaseof IL-10 producing cells in the patients and healthy individuals. Surprisingly, MSC-derivedexosomes demonstrated higher immune-modulating properties on conventional T cellsresponses, compared to mesenchymal stem cells (MSCs).

***
Conclusion:*** The current study, provides a novel approach of exocytosis on autoimmune therapy.In particular, Mesenchymal stem cell -derived exosomes, which are cell-derived biologics,could be considered as an alternative for Mesenchymal stem cells in treating MS.

## Introduction

Multiple sclerosis (MS) is a demyelinating autoimmune disorder of the central nervous system (CNS), where abnormal activation of particularly autoreactive T cells leads to neurodegeneration.^1^ Recent epidemiological studies have estimated 2.3 million of people around the world have MS^2^ and about 85% of these subjects demonstrated relapsing-remitting MS (RRMS) pattern.^3^ The exact cause of the disease is unknown and no absolute cure has been found yet. However, numerous biological and metaphysical modalities have been proposed to ameliorate the clinical scores of the MS,^4-6^ among which, mesenchymal stem cells (MSCs) demonstrated great promises for treating the disease.^7^

MSCs are multipotent stem cells with regulatory roles in immune responses.^8^ One of the underlying mechanisms behind their immunomodulatory function is the release of extracellular vesicles (EVs), particularly exosomes.^9^ Exosomes are nanoscale carriers, which their content (mostly proteins and small RNAs) is determined actively based on the transcriptome and proteome of the maternal cell. Under inflammatory conditions, for example, MSC-derived exosomes carry anti-inflammatory molecules (like transforming growth factor β [TGF-β], IL-10, and many microRNAs) to suppress the inflammation.^10^ Additionally, they are able to pass through boundaries like blood-brain barrier^11^; therefore, MSC-derived exosomes attracted attention to be used for the treatment of MS in several animal studies. In this case, intravenous (i.v.) administration of MSC-derived exosomes to the mouse models of MS, experimental autoimmune encephalomyelitis (EAE) and Theiler’s murine encephalomyelitis virus (TMEV)-induced demyelinating disease, have been shown to decrease the brain atrophy, T cell proliferation and their infiltration into the CNS, while elevated remyelination of damaged areas of the CNS and upregulated TGF-β, IL-4, and IL-10 in splenocytes.^12-14^ Moreover, MSC-derived exosomes were recently shown to decrease the proliferation of human peripheral blood mononuclear cells (PBMCs) as well as the levels of pro-inflammatory Th1 and Th17 cytokines in heathy subjects.^15^ However, *in vitro* or *in vivo* effects of MSC-derived exosomes have not been assessed on the MS subjects yet.

To elucidate the immunomodulatory potential of MSC-derived exosomes on MS patients, for the first time the present study aimed to evaluate suppression efficacy of both MSCs and MSC-derived exosomes on proliferation and cytokine production of the CD4^+^CD25^−^ conventional T cells (Tconv) isolated from RRMS patients and healthy controls.

## Materials and Methods

### Subjects

A total of 10 new cases of MS patients with a definite diagnosis of the disease, who were referred to the Iranian Center of Neurological Research at Imam Khomeini Hospital affiliated with Tehran University of Medical Sciences (TUMS) (6 Female, 4 Male; mean age: 35.00 ± 6.7 years; Expanded Disability Status Scale (EDSS): 2.89 ± 0.85), were included in this study (Table 1). The physical and neurological examinations were conducted by a neurologist according to the McDonald’s criteria.^16^ The use of disease-modifying treatments, including interferon-β (IFN-β) or any other immunomodulatory drugs was considered as exclusion criteria. All patients were clinically active and had symptoms at the time of sampling. Ten ethnically, age- and sex-matched healthy controls (6 Females, 4 males; age: 37.00 ± 5.5 years), who had no history of autoimmune or inflammatory diseases themselves and in their families, were also recruited into the present study. All participants signed a written informed consent and the study was approved by the Ethics Committee of Tehran University of Medical Sciences (TUMS, ethics code: IR.TUMS.VCR.REC.1397.1004).

**Table 1:** Demographic characteristics of RRMS patients

**Patient number**	**Sex**	**Disease** **Duration (month)**	**Age (year)**	**Phase (Relapse / Remission)**	**Physical activity (Assisted/Ambulant)**	**Symptoms**
P1	M	18	36	Relapse	Ambulant	Pain, numbness
P2	F	1	26	Relapse	Assisted	Pain, extreme weakness, balance disorder, diplopia
P3	M	12	26	Relapse	Ambulant	Pain, diplopia, numbness
P4	F	1	43	Relapse	Ambulant	Pain, fatigue
P5	M	1	29	Relapse	Ambulant	Fatigue, numbness
P6	F	3	44	Relapse	Assisted	pain, extreme weakness, balance disorder
P7	F	2	33	Relapse	Ambulant	Pain, fatigue
P8	M	12	43	Relapse	Ambulant	Pain, pins and needles
P9	F	24	37	Relapse	Ambulant	Pain, numbness
P10	F	12	35	Relapse	Ambulant	Pain, fatigue, diplopia

All patients were new cases of RRMS in relapse phase who never received any immunomodulatory medications in the past 3 to 6 months. Abbreviation: M: Male; F: Female

### Isolation, expansion, and characterization of umbilical cord tissue MSCs (UC-MSCs)

After having parents’ written consent, 38 to 40-week healthy umbilical cords (n = 20) were taken to the lab in phosphate buffer saline (PBS) supplemented with 300 μg/mL streptomycin and 300 U/mL penicillin (Gibco, Gaithersburg, MD, USA) at 2-8°C. Immediately afterwards, MSCs were isolated from discarded umbilical cords according to a standard protocol.^17^ In brief, the umbilical cords’ vein was washed with PBS, loaded with 0.1% (w/v) collagenase IV (Gibco, Gaithersburg, MD, USA), and incubated for 20 minutes at 37 °C and 5 % CO_2_. Afterward, the collagenase was quenched with Dulbecco’s modified Eagle’s medium/F-12 (DMEM/F-12) (Gibco, Gaithersburg, MD, USA) supplemented with 10 % fetal bovine serum (FBS), and the suspended cells were centrifuged at 500 × g for 5 minutes. Complete DMEM/F-12 containing 10% FBS, 4 mM L-glutamine, 100 μg/mL streptomycin, and 100 U/mL penicillin was then used to cultivate UC-MSCs at 37°C and 5 % CO_2_. The cells were evaluated for the expression of both positive and negative markers including HLA-DR-PerCP, CD11b-PE, CD14-PerCP-Cy5, CD34-FITC, CD45-APC, CD73-FITC, CD90-PE-Cy5, and CD105-PerCP (all from BioLegend, San Diego, CA, USA). Moreover, they were differentiated into the adipocytes and osteoblasts *in vitro*. To do so, MSCs were cultured in either adipogenic or osteogenic differentiation medium (Cyagen, Santa Clara, CA, USA) for 3 weeks. Then, MSCs were fixed by formaldehyde, and stained with oil red or alizarin red to identify lipid vacuoles or mineralization of calcium ions, respectively. Early passages of MSCs (2-4 passages) were used in all experiments unless otherwise stated.

To assess the proliferative capacity of stem cells isolated from different umbilical cords, the MSCs were harvested at the end of each passage and counted by trypan blue exclusion method. The number of cell population doubling (NCPD) and cell population doubling time (CPDT)^18^ were computed using the following equations:


$$NCPD=3.33\times \log\left(\frac{N_{t}}{N_{i}}\right)$$

$$CPDT=\left(t-t_{i}\right)\times \log\left\{2\times {\left[\log\left(\frac{N_{t}}{N_{i}}\right)\right]}^{-1}\right\}$$

Where N_t_ and N_i_ are the numbers of UC-MSCs at a specific time point t (day 15) and at initial seeding (day 0), respectively.

**Figure 1:**
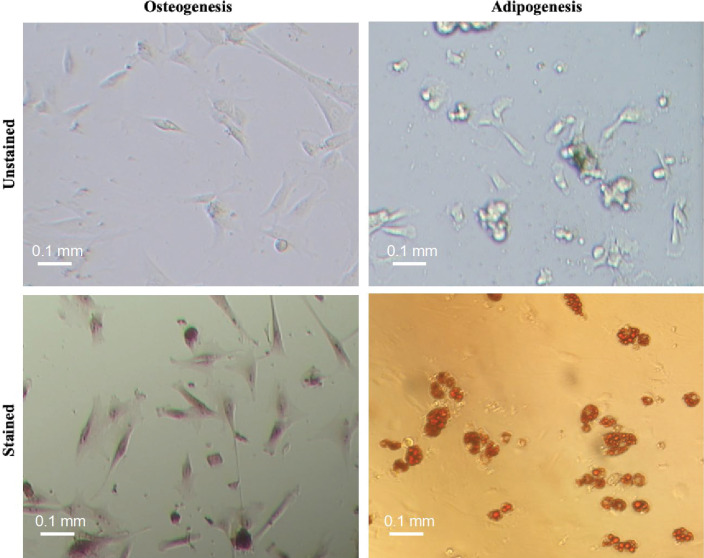
Osteogenic and adipogenic differentiation capacity of umbilical cord-derived-MSCs *in vitro*. To do so, MSCs were cultured at appropriate differentiation media for 3 weeks and then stained with Alizarin red and oil red to demonstrate intracellular accumulation of calcium (left) and generation of lipid vacuoles (right), respectively. The multipotency test was conducted for two umbilical cord derived cells and then the isolation protocol was scaled up for next isolations.

**Figure 2:**
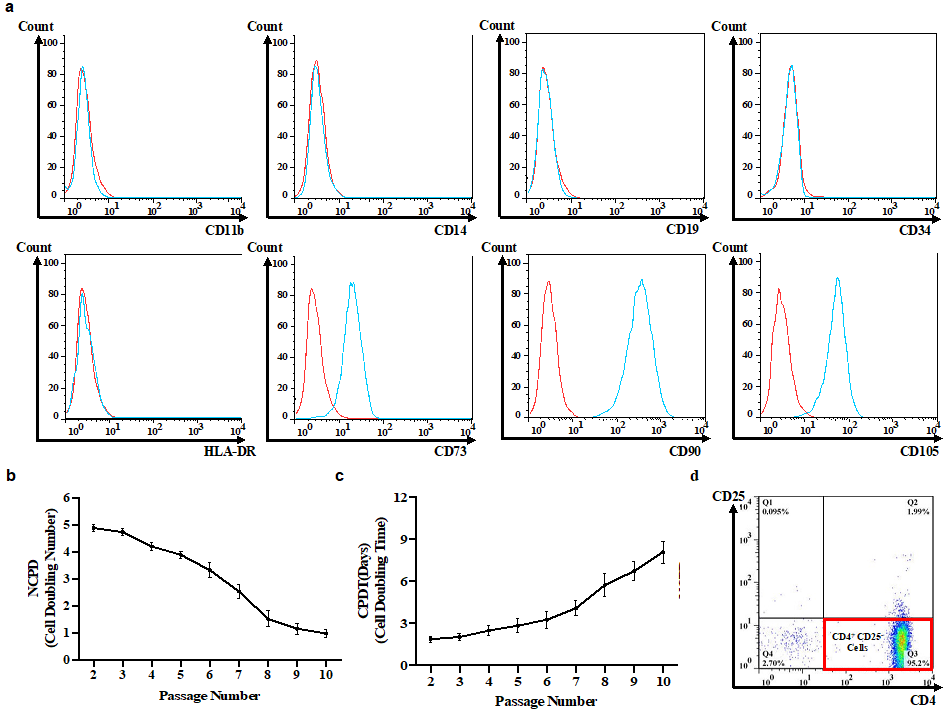
Characterization of *in vitro* isolated UC-MSCs and Tconvs. Flow cytometric immunophenotyping for surface markers of UC-MSCs (Red: unstained; blue: stained) (a). The Proliferative capability of UC-MSCs: The cell doubling rate (NCPD) decreased by 79.12 % from passage 3 to passage 10 (triplicate) (b) and thereafter, MSCs doubling time (CPDT) gradually increased over time (triplicate) (c). Immuopheotyping of conventional T cells were isolated from healthy individuals and RRMS subjects (duplicate) (d). UC-MSC, Umbilical cord tissue MSCs; Tconv, Conventional T cells; NCPD, Number of cell population doubling; CPDT, Cell population doubling time.

### Exosome isolation

To isolate exosomes, MSCs with 70% confluency were cultured with complete media containing 10% exosome-depleted FBS (System Biosciences, Palo Alto, CA, USA) for 72 hours. Conditioned media of cultured MSCs were collected and centrifuged at 300 × g for 5 minutes to eliminate the cells and debris. To remove microvesicles (large-sized extracellular vesicles), the supernatants were passed through a 0.22 µm filter and also centrifuged at 17000 × g for 30 min (Hitachi Koki himac CR22N Centrifuge) (NuAire, Lane Plymouth, MN, USA). The supernatants were then carefully isolated and centrifuged at 120000 × g for 90 minutes in an Optima XE-90-IVD Ultracentrifuge (Beckman Coulter, Carlsbad, CA, USA). Eventually, the exosome pellet was resuspended in PBS, dispensed into 100 µL aliquots, and stored at −80°C for further experiments. All centrifugations were taken at 4°C.

### Size characterization and quantification of exosomes

Each sample was individually diluted 1:10 in PBS to a total volume of 2 mL. Dynamic light scattering (DLS) measurements were conducted with ZetaSizer 3000-HA (Malvern Instruments, Malvern, UK), according to the standard settings (Refractive index = 1.331, viscosity = 0.89, temperature = 25°C).

To determine exosome concentration, we have used radioimmunoprecipitation assay (RIPA) buffer (Sigma-Aldrich, Gillingham, Dorset, UK) and protease inhibitor to lyse the MSC-derived exosomes according to the manufacturer’s protocol and then protein content of exosomes was indirectly quantified in a Bradford assay. To do so, 20 μL of each sample was added to 180 μL of Bradford reagent (Bio Rad Laboratories, Hercules, CA, USA) and then incubated at room temperature for 5 minutes. Absorbance level was read at 595 nm and the protein concentration of exosomes was extrapolated from a standard curve of bovine serum albumin.

The pellet provided by ultracentrifugation was also solubilized and fixed with 2.5% glutaraldehyde-PBS solution. The solution was then washed twice with PBS, and dehydrated by different concentrations of ethanol. Finally, the sample was left to dry and sent to investigate the morphology of exosomes using a scanning electron microscope (SEM) (Nova NanoSEM^TM^, Hillsboro, OR, USA).

### Purification of T cells and proliferation assay

Ficoll density gradient centrifugation (Lymphodex, Innotrain, Germany) method was used to isolate PBMCs. The CD4^+^CD25^−^ conventional T cells (Tconv) were purified using Dynabead Regulatory CD4^+^CD25^−^T cell kit, according to the manufacture’s guideline (Invitrogen, Waltham, MA, USA). To evaluate the purification yield, the cells were stained with CD4-APC and CD25-PE monoclonal antibodies (BioLegend, San Diego, CA USA), and were examined by BD FACSCalibur^TM^ flow cytometer (BD Biosciences, Heidelberg, Germany) and analyzed using FlowJo software.

The proliferation assay was conducted in 48 well plates. 1 × 10^6^ T cells were labeled with Cell Trace CFSE (BioLegend, San Diego, CA, USA), as stated in the manufacturer’s instruction. Then, T cells were cultured in the presence of either UC-MSCs (10:1 ratio) or 30 μg of MSC-derived exosomes.^19^ In addition, a well containing T cells without exosome or UC-MSC was also considered as untreated and positive control of proliferation for each sample. T cells were then polyclonally activated by 1 μg/mL of anti-CD3 and 5 μg/mL of anti-CD28 monoclonal antibodies (Mabtech, Stockholm, Sweden). Suppression index (S) was calculated based on the following equation:^20^

S = (a - b / a) × 100

where a is the percentage of proliferation in the absence of suppressor (MSC or MSC-derived exosome) and b is the percentage of proliferation in the presence of each suppressor alone.

Note that we have used an equal amount of the exosomes isolated from 1 × 10^5^ MSCs in T cell cultures.

### Intracellular cytokine assay

To analyze the immunomodulatory potential of the treatments on Th1, Th2, and Th17 cytokine production, the Tconv cells were cultivated in the presence of MSCs or their exosomes, followed by intracellular staining. In summary, differentiated cells were washed once with PBS and resuspended in 100 μl of staining buffer (PBS + 2% FBS). The cells were then fixed and permeabilized by LEUCOPERMTM (Bio-Rad, Hercules, CA, USA), according to the manufacturer’s manual. The antibodies including anti-IFN-γ-PE-Cy7, anti-IL-17A-PE (eBioscience, San Diego, CA, USA), and anti-IL-10-APC (BioLegend, San Diego, CA, USA) were used for staining and the cells were evaluated by flow cytometry.

### Statistical analysis

All statistical analyses were performed using R 3.6.1 software. The one-way ANOVA and Tukey’s post hoc tests were utilized to assess the statistical significance between the untreated groups and MSC- or MSC-derived exosome-treated group in RRMS patients and healthy controls (HC), respectively. Data are presented as means ± standard deviations (SD) unless otherwise stated, and two-sided *P* values < 0.05 were considered as statistically significant.

## Results and Discussion

The spindle-shaped cells were adherent to the cell culture flasks, capable of differentiating into the osteogenic as well as adipogenic cell lineage which is demonstrated by intracellular accumulation of calcium and lipid vacuoles, respectively (Figure 1). The cells were expressing CD73, CD90, and CD105 while were negative for HLA-DR, CD11b, CD14, CD34, and CD45 markers (Figure 2a). Extensive *in vitro* passage of MSCs isolated from various umbilical cords can potentially affect the properties of MSCs. We, therefore, monitored the doubling time of the cultivated cells in different passages to avoid early senescence of MSCs. Our results demonstrated a reduced proliferative rate of MSCs during sub-culturing (Figure 2b). In particular, the average NCPD gradually dropped behind after passage 3. In contrast, CPDT started to increase from 4.76 to 0.99 when the passage number enhanced from 3 to 10 (Figure 2c). Indeed, we decided to use early passages (2 and 3) of UC-MSCs in the next experiments. The purification yield of Tconvs was also evaluated after staining with anti-human CD4 and CD25 monoclonal antibodies and the FACS data demonstrated positive expression of CD4 and negative expression of CD25 (Figure 2d).

**Figure 3:**
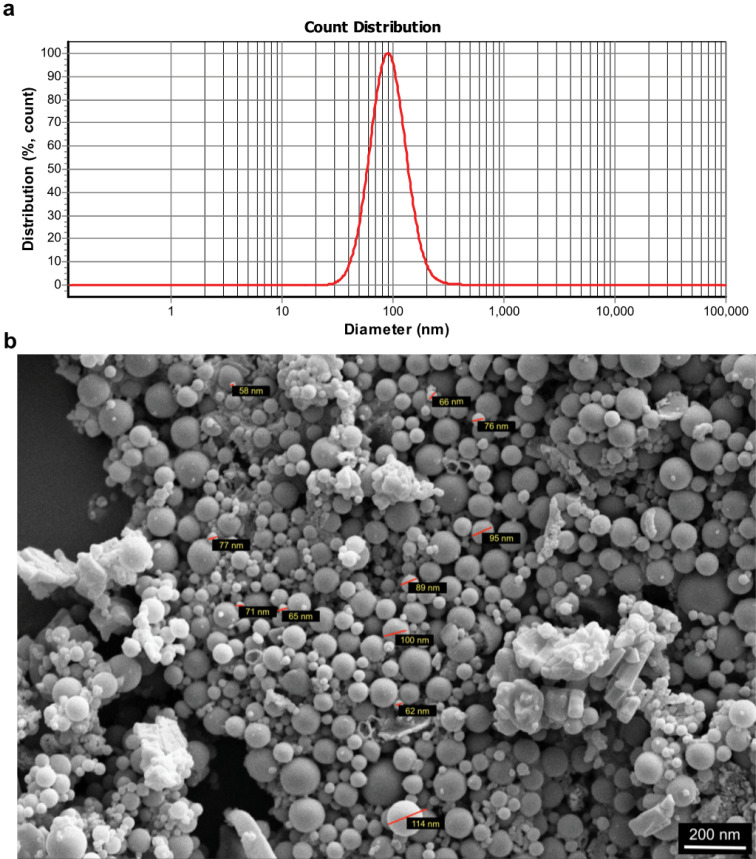
Characterization of UC-MSC-derived exosomes. Size distribution analysis by dynamic light scattering (DLS) (a); MSC-derived exosomes were first dispersed in phosphate buffer saline and then the samples were measured at 30 μg/mL concentration (the figure shows a representative line plot of one sample. All samples were assessed in duplicate). Morphology of UC-MSC-derived exosomes under a scanning electron microscope (SEM, 20000× magnification) (b).

**Figure 4:**
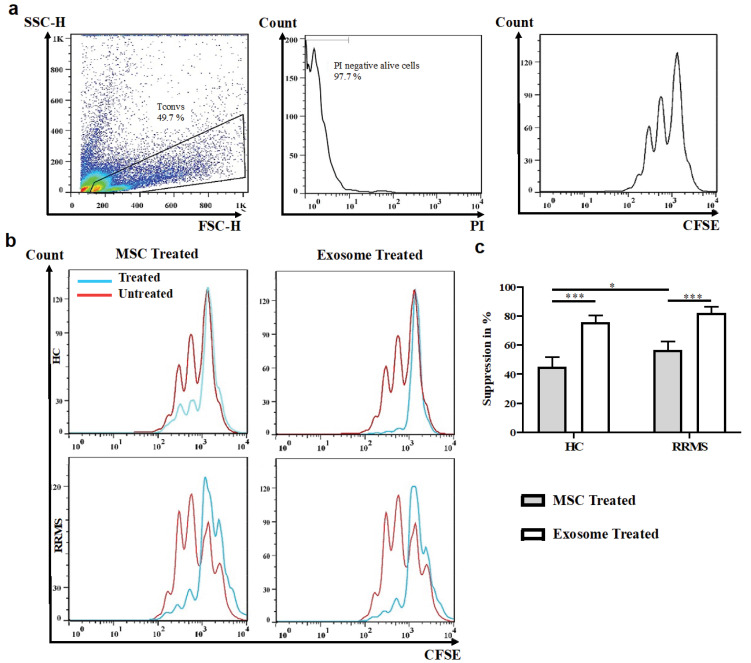
UC-MSC-derived exosomes exert immunosuppressive impact on *in vitro* proliferation of Tconvs. Human Tconvs were labeled with cell trace CFSE dye and polyclonally activated for 3 days in the absence or presence of MSCs or exosomes. Representative FACS plots of untreated healthy Tconvs (a) and MSC- or exosome- treated Tconvs (Red: untreated Tconvs; blue: MSC or exosome-treated Tconvs) (b) as well as its quantification (c). One-way ANOVA and Tukey’s post hoc were conducted to compare exosome treated Tconvs and MSC-treated Tconvs in healthy controls and RRMS patients. UC-MSC, Umbilical cord tissue MSCs; Tconv, Conventional T cells. *P* values less than 0.05, 0.01, and 0.001 are summarized with *, **, and ***, respectively. CFSE assay was conducted in duplicate.

Exosome size distribution assay indicated the exosomal average size of about 127 nm (Figure 3a). Then, one of aliquots was used to investigate size and morphology of the exosomes isolated from UC-MSCs under a scanning electron microscope (Figure 3b). Collectively, these data depicted that our protocols correctly isolated exosomes derived from UC-MSCs.

**Figure 5:**
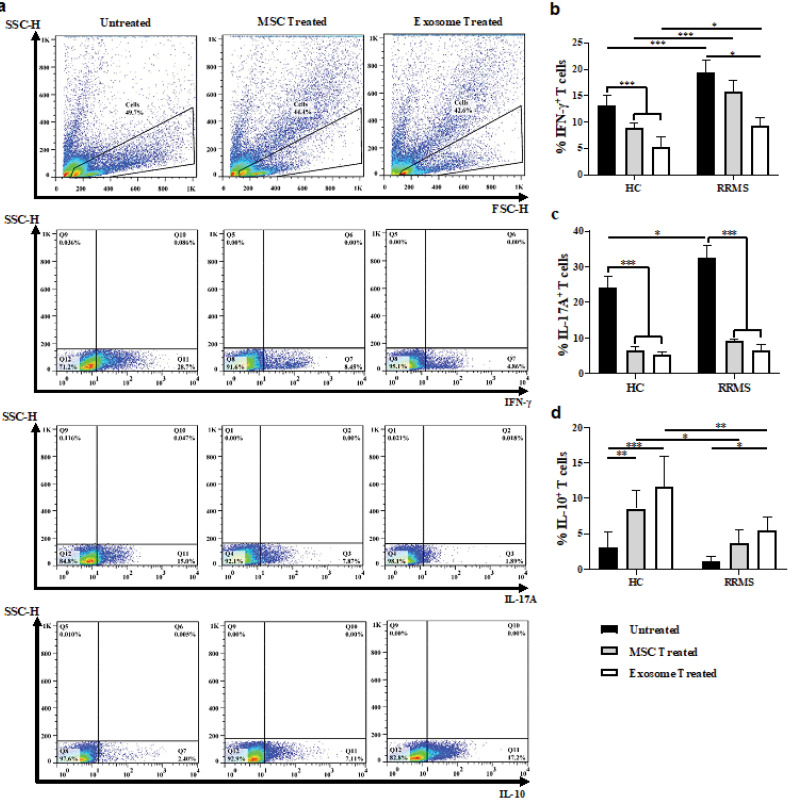
Induction of balance in the immune response of MSC- or exosome- treated Tconvs. Representative FACS plots of untreated and MSC- or exosome- treated Tconvs differentiated into IFNγ, IL-17A, or IL-10 producing T cells (a). Quantification of percentage reduction/increase of IFNγ, IL-17A, or IL-10 producing T cells in the absence or presence of MSC or MSC-derived exosomes (b-d).

One-way ANOVA and Tukey’s post hoc were conducted to compare between untreated and exosome- or MSC- treated Tconvs in healthy controls and RRMS patients. UC-MSC, Umbilical cord tissue MSCs; Tconv, Conventional T cells. *P* values less than 0.05, 0.01, and 0.001 are summarized with *, **, and ***, respectively. Intracellular stainings were performed with no replicate.

During MS pathogenesis, conventional T cells proliferate and differentiate into autoreactive lymphocytes in response to self-antigens. As an advantage, exosomes were generally shown to have less immunogenicity than cell therapy in allogenic settings.^21,22^ We, thus, sought to primarily characterize the suppressive impacts of MSCs and their exosomes on conventional T cell proliferation. Flow cytometry analyses of proliferated cells demonstrated higher suppression index (S) for exosome-treated Tconv cells in patients and healthy controls (S_exo-P_ = 82.29 ± 5.83; S_exo-HC_ = 75.61 ± 4.97; *P* = 0.25) than that for MSC-treated T cells (S_MSC-P_ = 56.59 ± 6.37; S_MSC-HC_ = 45.15 ± 6.48; *P* = 0.02) (Figure 4 a-c). Moreover, exosomes depicted higher suppression capacity than MSCs in both healthy controls (*P* < 0.001) and RRMS patients (*P* < 0.001). This result is in line with a previous study that demonstrated that MSC-derived exosomes exert a slightly higher anti-inflammatory impact on T cell proliferation.^23^ Two studies have also revealed that MSC-derived exosomes successfully suppressed the proliferation of healthy PBMCs as well as CD4^+^/CD8^+^ T cells and decreased the amount of IL-12, IL-6, IL-17, and IL22 produced by activated PBMCs.^15,24^ A great number of other studies have examined total extracellular vesicles (microvesicles and exosomes) derived from human MSCs in allogenic settings and reported efficient suppression on the proliferation of B and NK cells, although no or low functional impacts were observed on T cell proliferation.^10,25,26^ Besides, some animal studies exhibited that human MSC-derived exosomes failed to inhibit Con A-activated splenocytes and T cells in mouse and rat, respectively. However, these investigations indicated that MSC-derived exosomes activate MYD88-dependent signaling through Toll-like receptor 4 ligands which leads to M2 macrophage phenotype concomitant with increasing CD4^+^CD25^+^FoxP3^+^ regulatory T cell polarization.^27,28^

The conflicting outcomes regarding proliferation suppression might be due the various sources of MSCs, culture conditions, isolation protocols, infectivity of the xenogeneic exosomes on the recipient organism, and the use of freeze and thawed exosomes.

Next, we reasoned whether MSC-derived exosomes change the cytokine production profiles of pathogenic T cells in the healthy subjects and patients. Indeed, we stained Tconv cells for IFN-γ, IL-10, and IL-17A after treatment with either MSCs or their exosomes (Figure 5a). Both treatments exhibited a comparable efficacy with respect to decreasing in the percentage of IFN-γ (Figure 5b) and IL-17 (Figure 5c) producing cells as well as elevating IL-10 producing cell frequency (Figure 4d); Surprisingly, the intervention also showed some major differences between patients and control group with similar treatments (Figure 5b-d). Other studies have also found MSC-derived exosome capable of suppressing the differentiation of IFN-γ and IL-17 producing T cells^29,30^; whereas at protein level, IL-10 was shown to substantially increase in the exosome-treated leukocytes.^31^ That is, allogenic MSCs and MSC-derived exosomes can enormously enhance regulatory cell (regulatory T cells, Th 2 cells etc) functions in order to balance the impaired immune response in MS. To further elucidate how MSC-derived exosomes suppress peripheral inflammation, unraveling the other molecular content of exosomes may explain its potential for our observed results. In this respect, proteomic analysis of MSC-derived exosomes demonstrated high concentration of peptides including galectin-1, macrophage inhibitory cytokine 1, latent-transforming growth factor β-binding protein, and heat shock protein 70.^15^ MSC-derived vesicles were also shown to carry PD-L1, galecin-1, and TGF-β, and therefore were successfully applied in the treatment of EAE.^32^ Moreover, numerous other investigations found T cell response suppressors including TGF-β, IL-10, prostaglandin-E2, miR-155, miR-146a, miR-181c, miR-17, miR-21, and many other factors within MSC-derived exosomes which suppress T cell response.^33^ These molecules were found by investigations that considered that MSC-derived vesicles are capable of transferring anti-inflammatory molecules into the autoreactive cells and re-inducing a self-tolerance.

## Conclusion

In the present study, for the first time we depicted that human UC-MSC-derived exosomes mimic the immunomodulatory benefits of their parental cells and can more effectively suppress the proliferation and pathogenic function of the Tconvs in both RRMS patients and healthy controls. These findings introduce MSC-derived exosomes as an alternative to therapeutic MSCs, providing a novel approach for treating MS. However, considerable issues remain to be resolved and further investigations should be done to validate the efficacy of MSCs as well as their exosomes in large-scale applications.

## Acknowledgments

We are thankful to the Department of Immunology facilities at Tehran University of Medical Sciences.

## Ethical Issues

The present study was conducted with approval from the Tehran University of Medical Sciences (TUMS), Tehran, Iran.

## Conflict of Interest

The authors declare that they have no competing interests.
